# Possible Changes of the Future

**Published:** 1916-01-29

**Authors:** 


### POSSIBLE CHANGES OF THE FUTURE.

Though, unhappily, no responsible authority can pretend to foresee even an approximate date for the termination of the war, the problems which will follow this event, or some of them at least, are not escaping recognition and attention. Probably many questions which will then arise are beyond the anticipation even of the wisest, for history, and even quite recent history, has not crowned with a high measure of success the prognostic efforts either of the amateur prophet or of the experienced statesman. Still, some things can be counted on, and it is well to be prepared for peace just as we are now learning in a severe school that it would have been well had we been prepared for war. Though the unexpected, as is its wont, is very likely indeed to happen, there is no reason for refusing to arrange our plans with a view to meet what circumstances suggest as probable. Time and the event may in some measure contradict our judgment, and unanticipated emergencies may upset our schemes, but even with these contingencies in view no wise man will counsel an absolute adherence to the policy of the famous phrase which bids us " wait and see." In some respects, doubtless, there is no alternative, but many others are open to intelligent preparation. There are just now a number of activities afoot which show that this truth is realised, and indeed, next to the stern determination to go on to a complete success, however great the sacrifice, the thought of organisation for post-war conditions is beginning to occupy the attention of the country.

Prominent amongst the changes which the future is likely to bring is a more serious examination of our educational methods. The military efficiency which has marked the activities of the enemy is by some regarded as a proof of the soundness of the national policy in respect to the training and development of the individual citizen, and it is urged that this example ought not to be lost on ourselves. Just now, and for obvious reasons, the persons who uphold this conclusion do not voice it very loudly, but we have only to recall many exhortations addressed to us in recent years to feel sure that in due time the same policy will reappear. On the other hand are those who would fain have us believe that all the horrors of the European position may be traced back almost entirely to a pernicious scheme of education, and that the vaunted triumphs in the world of intellect to which we formerly paid much deference owe their plumes to dexterous and unblushing theft. Between such conflicting counsels the plain man may readily halt in uncertainty. But what is to be hoped is that the contradiction will arouse a conviction of the importance of education in the scheme of national life, and will secure a determination to afford it the attention and support which are its due. In the end it is ideas which carry the day, and this is evident even amidst the clash of arms. How necessary are these lessons is emphasised by certain proposals to cut down the national expenditure, both on education and on scientific research. In other words we are to diminish our fitness for the economic struggle which both experience of the past and the obvious intimations of the present hour announce plainly to be an inevitable feature of modern civilisation. It is difficult enough to accompany the optimists who believe that the present war is really to be the last of the long series of combats which have marked the history of the world, but still more difficult is it to go with those who think that when the fighting has ceased a sense of international fraternity and goodwill is promptly to appear. To accept such a position is surely to close one's eyes to patent facts. Whether for good or ill, scientific knowledge announces itself confidently as a condition of success in the world of affairs, and more and more it will dominate economic and commercial progress even as to-day it rules the art of war and the stricken field. To our readers comes nearer the reflection that even in a more conspicuous degree does medicine rely on science, and that on the application of the latter depends the health both of our armies and of the community generally. Hence it is appropriate here to make a special appeal for a public opinion in opposition to movements which by the practice of small, immediate economies would put in peril the large national interest of the public health.

Yet the case for science may perhaps be overstated, or at least it may be stated with forgetfulness of other claims. There are those who are not content to press its educational values unless at the same time they may disparage other influences. From a recent public correspondence the conclusion might be reached that a scientific training is the only educational discipline which is worthy of encouragement and preservation. There will be a long movement before such a proposition receives general acceptance in this country. Whatever our national defects may be we have at least bred men as worthy of recognition as those of the spacious past; and the methods which have done this will not be lightly cast aside. As in the general world, so in medicine, there is room for a training which is not limited to rigid technicalities, and for our own part we should be sorry to see a hurried desire to cast aside influences which have helped to make the medical profession something more than a mere school of clever and dexterous craftsmen.

